# Metabolic Syndrome and Hand Osteoarthritis in Postmenopausal Women: Association With Disease Severity and Bone Mineral Density

**DOI:** 10.7759/cureus.117466

**Published:** 2026-10-05

**Authors:** Siham Rachidi, Saadia Ait Malek, Erraoui Mariam, Imad Ghozlani

**Affiliations:** 1 Rheumatology, University Hospital of Mohammed VI, Agadir, MAR; 2 Rheumatology, Cartilage and Bone (CARBONE) Research Team, Research Laboratory of Innovation in Health Sciences (LARISS) Faculty of Medicine and Pharmacy of Agadir, Ibn Zohr University, Agadir, MAR; 3 Rheumatology, Oued Eddahab Military Hospital, Agadir, MAR

**Keywords:** bone density, hand osteoarthritis, metabolic syndrome (metsy), morocco, post menopausal women

## Abstract

Introduction: Hand osteoarthritis (HOA) is highly prevalent in postmenopausal women and may reflect an underlying metabolic disorder. Chronic low-grade inflammation driven by obesity, hyperglycemia, and estrogen deficiency may contribute to this metabolic phenotype. This study aimed to investigate the association between metabolic syndrome (MetS), its individual components, and HOA, and to explore its relationship with bone mineral density (BMD).

Methods: We conducted a retrospective observational study, with descriptive and analytical components, including 252 postmenopausal women aged ≥50 years presenting with hand pain. MetS was defined according to the National Cholesterol Education Program Adult Treatment Panel III (NCEP-ATP III) criteria. HOA was assessed clinically and radiographically using the Kellgren-Lawrence (K&L) grading system. BMD was measured using dual-energy X-ray absorptiometry (DEXA), and vertebral fractures were evaluated using vertebral fracture assessment (VFA).

Results: The mean age of participants was 62.6 ± 7.3 years, with a mean body mass index (BMI) of 32.3 ± 6.5 kg/m². The prevalence of symptomatic HOA was 28.3%, while radiographic HOA was observed in 19.1% of patients. MetS was present in 30.3% (n=76) of the cohort. HOA was significantly associated with advanced age, higher BMI, and MetS (p < 0.001). Radiographic severity of HOA increased significantly with age, body weight, duration of menopause, and metabolic abnormalities. Increased severity was also associated with lower BMD and a higher prevalence of vertebral fractures (p < 0.001). In multivariate analysis, MetS was independently associated with symptomatic HOA (OR 4.3; 95% CI: 1.92-9.12; p < 0.001), along with hypertension. While no overall association was found between HOA and BMD, lower BMD and vertebral fractures were significantly associated with increased radiographic severity of HOA.

Conclusion: HOA in postmenopausal women is strongly associated with MetS, supporting the concept of a metabolic phenotype of osteoarthritis. These findings emphasize the importance of comprehensive cardiometabolic risk assessment in the management of HOA and suggest a link between disease severity and bone fragility. As the first study of its kind conducted in Morocco, further large-scale multicenter studies are needed to confirm these findings.

## Introduction

Osteoarthritis (OA) is the most prevalent musculoskeletal disorder worldwide and a leading cause of pain and disability among older adults. According to the World Health Organization, approximately 9.6% of men and 18% of women aged 60 years or older have symptomatic OA. The disease is characterized by progressive cartilage degradation, subchondral bone remodeling and osteophyte formation, reflecting an imbalance between catabolic and anabolic processes within the joint [1].

Hand osteoarthritis (HOA) is one of the most common peripheral localizations, particularly in postmenopausal women. Its prevalence has been reported to range from 37.7% in men to 44.2% in women [2]. Beyond mechanical factors, increasing evidence suggests a systemic component linking OA to metabolic abnormalities, leading to the concept of “metabolic osteoarthritis”. Several biological pathways may explain this association, including chronic low-grade inflammation mediated by adipokines, oxidative stress induced by hyperglycemia, and insulin resistance [3].

Metabolic syndrome (MetS), defined by the presence of at least three metabolic abnormalities, including central obesity, hypertension, dyslipidemia, and impaired glucose metabolism, has been increasingly associated with OA, with a prevalence of MetS reported in up to 59% among patients with OA [4].

However, epidemiological data on HOA and its metabolic associations remain limited in North African populations. Therefore, this retrospective study aimed to determine the prevalence of HOA in postmenopausal women and to evaluate its association with MetS and its individual components, as well as its relationship with bone mineral density (BMD).

## Materials and methods

Study design and population

This was a retrospective observational study with descriptive and analytical components and was conducted in the Rheumatology Department of Oued Eddahab Military Hospital between January 2013 and December 2024. The study included postmenopausal women aged ≥50 years presenting with hand pain.

As this was a retrospective study, no formal a priori sample size or statistical power calculation was performed. The final sample consisted of all eligible patients identified during the study period. A total of 252 patients were included after applying predefined inclusion and exclusion criteria.

Inclusion and exclusion criteria

Inclusion and exclusion criteria are shown in Table 1.

**Table 1:** Inclusion and Exclusion Criteria HOA: hand osteoarthritis

Category	Criteria
Inclusion criteria	Postmenopausal women aged ≥50 years presenting with hand pain
Exclusion criteria	Chronic inflammatory rheumatic diseases (e.g., rheumatoid arthritis, psoriatic arthritis) or crystal-induced arthropathies
	Systemic autoimmune diseases
	Secondary osteoarthritis related to infection or acute trauma
	History of hand surgery or hand fractures
	Other hand conditions interfering with HOA assessment (De Quervain’s tenosynovitis, trigger finger, Dupuytren’s disease, synovial chondromatosis, carpal tunnel syndrome)

Data collection and study variables

Collected variables included demographic characteristics, particularly age, as well as anthropometric parameters such as body mass index (BMI) and waist circumference. Metabolic parameters were also assessed, including fasting plasma glucose, triglycerides levels, high-density lipoprotein (HDL) cholesterol, and blood pressure. In addition, radiographic features of HOA were evaluated, along with BMD measurement. The presence of vertebral fractures was also systematically recorded. 

Radiographic assessment of hand osteoarthritis

Standardized posteroanterior radiographs of both hands and wrists were obtained. All radiographs were assessed by a single experienced reader using the Kellgren-Lawrence (K&L) grading system [5] to determine the presence and severity of radiographic HOA.

Sixteen joints per hand were evaluated, including distal interphalangeal (DIP), proximal interphalangeal (PIP), metacarpophalangeal (MCP), thumb interphalangeal, first carpometacarpal (CMC1), and scaphotrapeziotrapezoidal (STT) joints. Radiographic HOA was defined as K&L grade ≥2.

For patient-level severity classification, the overall K&L grade was defined according to the highest K&L grade observed among the assessed joints of both hands. Thus, each participant was assigned a grade ranging from 0 to 4 according to the maximum radiographic severity observed.

Interobserver reproducibility was assessed on 50 radiographs by a second independent reader, yielding a mean kappa coefficient of 0.77 and an agreement rate of 94%. The primary reader's assessment was performed blinded to the patients' clinical and metabolic characteristics.

Assessment of symptomatic hand osteoarthritis

Symptomatic HOA was defined by the presence of at least one of the clinical feature, including mechanical hand pain with a visual analog scale (VAS) score greater than 10 mm, joint stiffness, or functional limitation affecting daily hand activities

Definition of metabolic syndrome

MetS was defined according to the National Cholesterol Education Program Adult Treatment Panel III (NCEP-ATP III) criteria [6] by the presence of at least three of the following components: waist circumference ≥88 cm, blood pressure ≥130/85 mmHg or current use of antihypertensive treatment, fasting plasma glucose ≥110 mg/dL or previously diagnosed diabetes, triglycerides ≥150 mg/dL, and HDL cholesterol <50 mg/dL. Total body fat mass was assessed using dual-energy X-ray absorptiometry (DEXA).

Bone mineral density and vertebral fractures

BMD was measured at the lumbar spine and femoral neck using a Hologic DEXA system (Hologic, Inc., Marlborough, MA, USA). Osteoporosis was defined as a T-score ≤ -2.5, and osteopenia as a T-score between -2.5 and −1. Vertebral fractures were assessed using vertebral fracture assessment (VFA) and classified according to the semi-quantitative Genant method.

Anthropometric and biological measurements

Waist circumference, BMI, and blood pressure were measured using standardized protocols. Physical activity level was categorized as low, moderate, or high. Fasting blood samples were collected to measure triglycerides, HDL cholesterol, and glucose levels.

Ethical considerations

The study was conducted in accordance with the principles of the Declaration of Helsinki and was approved by the Ethics Committee of the Faculty of Medicine and Pharmacy of Marrakech, Morocco. At the time of the study, no formal approval number was issued for this type of observational research. Written informed consent was obtained from all participants.

Statistical analysis

Statistical analysis was performed using SPSS version 20.0 (IBM Corp., Armonk, NY, USA). Continuous variables were expressed as mean ± standard deviation (SD), while categorical variables were presented as frequencies and percentages. Comparisons between groups (with and without HOA) were performed using the chi-square test for categorical variables and Student’s t-test for continuous variables. For comparisons of continuous variables across the different K&L radiographic severity grades, ANOVA was used, as appropriate. 

Variables with p < 0.20 in univariate analysis were included in a multivariate logistic regression model to identify independent risk factors associated with symptomatic HOA. Results were reported as odds ratios (ORs) with 95% confidence intervals (CIs). A p-value ≤ 0.05 was considered statistically significant.

## Results

Descriptive analysis

Demographic Characteristics

A total of 252 postmenopausal women were included in the study. The mean age was 62.6 ± 7.3 years (range: 50-90 years), with a predominance of patients aged 60-69 years (42.4%, n=107). The mean BMI was 32.3 ± 6.5 kg/m², and the mean duration of menopause was 12.6 ± 8.1 years. Detailed demographic characteristics are summarized in Table 2.

**Table 2:** Characteristics of the Study Population BMI: body mass index, BMD: bone mineral density

Variable	n (%)	mean ± SD
Age (years)		62.6 ± 7.3
Age group 60-69 years	107 (42.4%)	
BMI (kg/m²)		32.3 ± 6.5
Duration of menopause (years)		12.6 ± 8.1
Metabolic syndrome	76 (30.3%)	
Waist circumference (cm)		104.3 ± 10.4
Hip circumference (cm)		113.3 ± 13.4
Total body fat (%)		47.2 ± 4.8
Diabetes	85 (33.9%)	
Hypertension	103 (41%)	
Dyslipidemia	73 (29.1%)	
Lumbar spine BMD (g/cm²)		0.97 ± 0.1
Femoral neck BMD (g/cm²)		0.84 ± 0.1
Lumbar T-score		-1.5 ± 1.3
Femoral T-score		-1.4 ± 1
Osteoporosis	80 (31.9%)	
Vertebral fractures	36 (14.3%)	

Metabolic Syndrome and Its Components

MetS, as defined by the NCEP-ATP III criteria, was present in 76 patients (30.3%). The mean waist circumference was 104.3 ± 10.4 cm, and the mean hip circumference was 113.3 ± 13.4 cm. The mean total body fat percentage was 47.2 ± 4.8%. Regarding individual components of MetS, diabetes was present in 33.9% (n=85) of patients, hypertension in 41% (n=103), and dyslipidemia in 29.1% (n=73) (Table 2).

Bone Mineral Density and Osteoporosis

The mean BMD was 0.97 ± 0.1 g/cm² at the lumbar spine and 0.84 ± 0.1 g/cm² at the femoral neck. The mean T-scores were -1.5 ± 1.3 at the lumbar spine and -1.4 ± 1 at the femoral neck.

According to WHO criteria, osteoporosis was diagnosed in 80 patients (31.9%). Vertebral fractures (Grades 2 and 3), assessed using VFA and classified according to the Genant method, were observed in 36 patients (14.3%) (Table 2).

Hand Osteoarthritis

Symptomatic HOA was identified in 71 patients, corresponding to a prevalence of 28.3%. Radiographic HOA (K&L grade ≥2) was present in 48 patients (19.1%). The distribution of K&L grades was as follows: grade 0 (70%), grade 1 (10.8%, n=27), grade 2 (11.2%, n=28), grade 3 (6.4%, n=16), grade 4 (1.6%, n=4) (Figure 1). 

**Figure 1:**
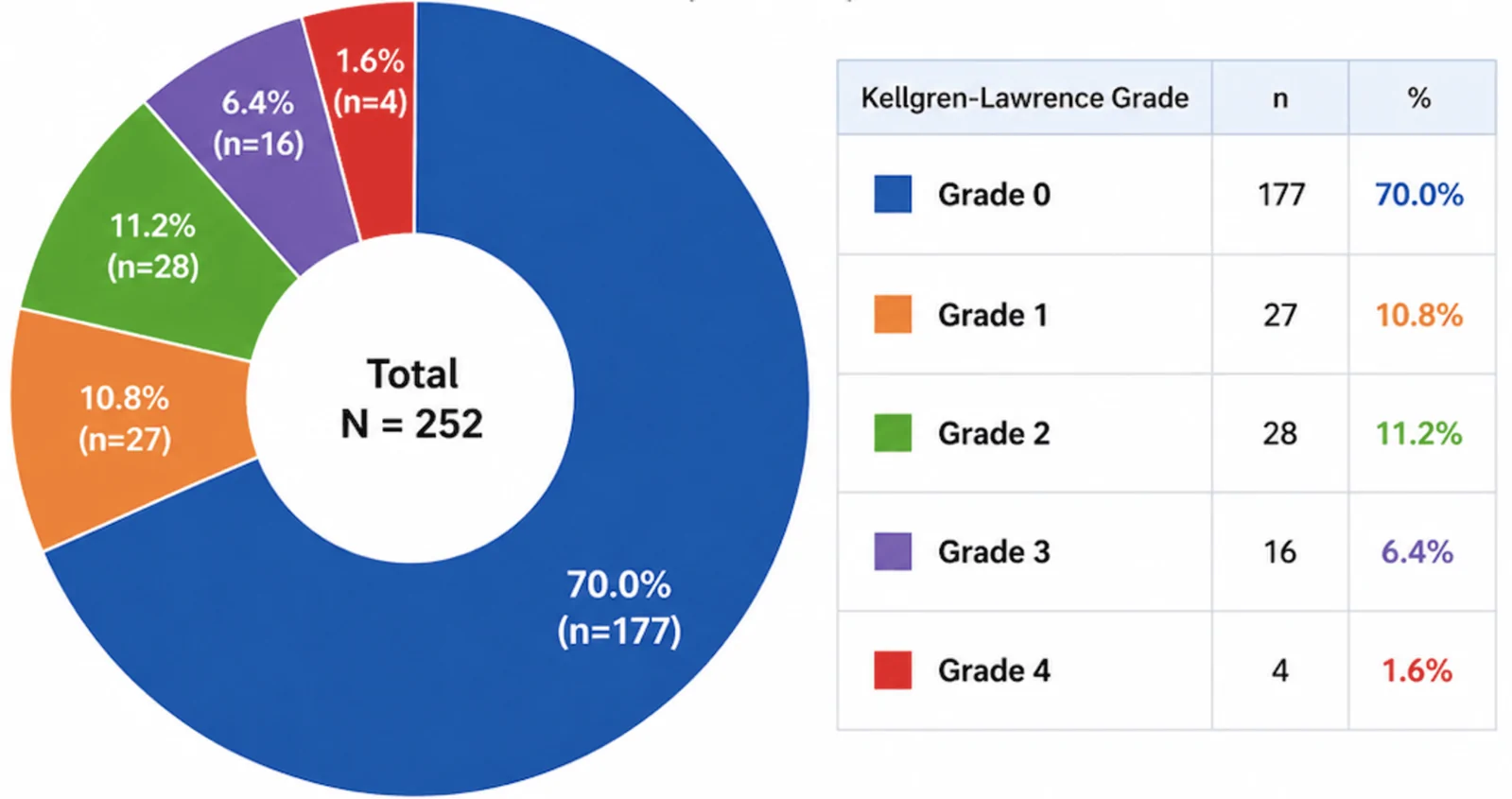
Distribution of Kellgren-Lawrence grades among the study population (N=252)

Analytical analysis

Association Between Demographic Characteristics and HOA

Patients with symptomatic HOA were significantly older than those without HOA (66.7 ± 6.5 versus 61.6 ± 8.7 years, p < 0.001). They also had a significantly higher mean body weight (81.4 ± 10.8 kg versus 73.8 ± 11.6 kg, p < 0.001) and BMI (34.5 ± 5.5 kg/m² versus 31.7 ± 6.6 kg/m², p = 0.005). No significant differences were observed regarding height, duration of menopause, parity, or physical activity level (Table 3).

**Table 3:** Comparison of clinical, metabolic, and bone parameters according to the presence of symptomatic hand osteoarthritis Values are expressed as mean ± standard deviation or percentages HOA: hand osteoarthritis, BMI: body mass index, BMD: bone mineral density, NS: not significant

Variable	Symptomatic HOA (N=71)	No HOA (N=181)	P-value
Age (years)	66.7 ± 6.5	61.6 ± 8.7	<0.001
Weight (kg)	81.4 ± 10.8	73.8 ± 11.6	<0.001
BMI (kg/m²)	34.5 ± 5.5	31.7 ± 6.6	0.005
Metabolic syndrome n(%)	42 (59.2)	34 (18.9)	<0.001
Diabetes n(%)	34 (47.9)	51 (28.3)	0.03
Hypertension n(%)	48 (67.6)	55 (30.6)	<0.001
Dyslipidemia n(%)	31 (43.7)	42 (23.3)	0.001
Total body fat (%)	49.8±4.5	46.4±4.7	<0,001
Lumbar spine BMD (g/cm²)	1±0.1	0.966±0.1	NS
Femoral neck BMD (g/cm²)	0.845±0.1	0.841±0.1	NS
Osteoporosis n(%)	16 (22.5)	48 (26.7)	NS
Vertebral fractures n(%)	16 (23.5)	20 (12.2)	NS

Association Between Metabolic Syndrome and Symptomatic HOA

The prevalence of MetS was significantly higher in patients with symptomatic HOA compared to those without HOA (59.2%, n = 42 versus 18.9%, n = 34; χ² = 37.22, df = 1, p < 0.001, Cramér’s V = 0.39).

Diabetes was more frequent in patients with symptomatic HOA (47.9%, n = 34 versus 28.3%, n = 51; χ² = 4.63, df = 1, p = 0.03, Cramér’s V = 0.14). Similarly, hypertension (67.6%, n = 48 versus 30.6%, n = 55; χ² = 27.37, df = 1, p < 0.001, Cramér’s V = 0.33) and dyslipidemia (43.7%, n = 31 versus 23.3%, n = 42; χ² = 10.75, df = 1, p = 0.001, Cramér’s V = 0.21) were significantly more prevalent in the symptomatic HOA group.

Patients with symptomatic HOA also had significantly higher mean waist circumference (109.1 ± 9.4 cm versus 102.9 ± 10.3 cm, p < 0.001), hip circumference (118.7 ± 18.4 cm versus 111.8 ± 11.2 cm, p = 0.001), and total body fat percentage (49.8 ± 4.5% versus 46.4 ± 4.7%, p < 0.001) (Table 3).

Bone Mineral Density and Symptomatic HOA

No significant differences were observed between patients with and without symptomatic HOA in terms of BMD at the lumbar spine (1 ± 0.1 g/cm² versus 0.966 ± 0.1) or femoral neck (0.845 ± 0.1 g/cm² versus 0.841 ± 0.1). Similarly, T-scores at both sites were comparable between groups. There were no significant differences in the prevalence of osteoporosis (22.5%, n = 16 versus 26.7%, n = 48) or vertebral fractures (23.5%, n = 16 versus 12.2%, n = 20) (Table 3).

Factors Associated With Radiographic Severity of HOA

A significant increase in mean age was observed with increasing radiographic severity of HOA, ranging from 63.2 ± 6.5 years (grade 2) to 78 ± 9.9 years (grade 4) (p < 0.001). Mean body weight, duration of menopause, and number of pregnancies also increased significantly with HOA severity (p < 0.001, p < 0.001, and p = 0.002, respectively), whereas no association was found with height or BMI.

Regarding metabolic factors, the prevalence of MetS, hypertension, and diabetes increased significantly with HOA severity (p < 0.001, p = 0.002, and p = 0.02, respectively). Waist circumference, hip circumference, and total body fat percentage also showed significant variation across radiographic grades (p = 0.02, p = 0.04, and p = 0.002, respectively). No significant association was found with dyslipidemia.

Bone Mineral Density and HOA Severity

A significant decrease in both lumbar and femoral BMD was observed with increasing radiographic severity of HOA (p = 0.01 and p = 0.004, respectively). Lumbar and femoral T-scores also varied significantly according to K&L grades (p = 0.02 and p < 0.001, respectively). The presence of grade 2 or 3 vertebral fractures was significantly associated with HOA severity (p < 0.001), whereas no association was found with overall osteoporosis prevalence (p = 0.13).

Multivariate Analysis

Multivariate logistic regression analysis identified MetS and hypertension as independent factors associated with symptomatic HOA. MetS was associated with higher odds of symptomatic HOA (OR 4.3; 95% CI: 1.92-9.12; p < 0.001). Hypertension was also independently associated with symptomatic HOA (OR 2.1; 95% CI: 1.06-5.01; p =0,035) (Table 4).

**Table 4:** Multivariate Analysis

Variable	OR	95%CI	P-value
Metabolic syndrome	4.3	1.92-9.12	<0.001
Hypertension	2.1	1.06-5.01	0,035

## Discussion

Hand osteoarthritis

HOA is one of the most common forms of OA, predominantly affecting postmenopausal women. It is characterized by structural joint changes including cartilage degradation, subchondral bone remodeling, osteophyte formation, and varying degrees of synovial inflammation. Clinically, HOA presents with pain, stiffness, functional limitation, and characteristic nodal deformities such as Heberden’s and Bouchard’s nodes, which may significantly impair quality of life [7].

Epidemiology

The prevalence of HOA varies widely across studies depending on population characteristics and diagnostic criteria. Large epidemiological studies such as the Framingham [2] and Rotterdam [8] cohorts have reported high rates of radiographic HOA, particularly in older populations, with prevalence of respectively 38.2% and 56%.

In our study, the prevalence of radiographic HOA (defined as K&L grade ≥2 in at least one joint) was 19.1%, which is lower than that reported in Western cohorts. This discrepancy may be explained by differences in study design, age distribution, and ethnic factors.

Risk factors for hand osteoarthritis

Non-Modifiable Factors

Age is the most consistent risk factor for HOA, with both prevalence and severity increasing markedly after the age of 50. Female sex is also a major determinant, with a higher incidence observed in women, with a relative risk of approximately 1.23 for prevalence and 1.54 for incidence, particularly during the perimenopausal period, supporting a key role of estrogen deficiency in disease pathophysiology [9].

The relationship between BMD and HOA remains controversial. Marcelli et al. [10] reported that higher HOA scores were associated with higher BMD at various skeletal sites, whereas other studies, such as the Rancho Bernardo study [11], reported reduced BMD in women with HOA, particularly in severe forms.

In our study, although no overall association was observed between symptomatic HOA and BMD, lower BMD and vertebral fractures were associated with greater radiographic severity of HOA. These findings support a complex relationship between OA and bone metabolism. However, given the nature of the analysis, causal relationships cannot be inferred. 

Genetic factors also contribute significantly. Twin heritability estimates of approximately 60% and evidence from genome-wide association studies (GWAS) have identified several candidate genes involved in growth factor signaling, extracellular matrix integrity and calcification, and inflammatory pathways [12].

Modifiable Factors

Obesity is the most robust modifiable risk factor for HOA, with strong evidence linking adiposity to both disease occurrence and severity [13]. This association is not only mechanical but also mediated by systemic metabolic and inflammatory pathways, particularly involving adipokines.

Occupational exposure and repetitive mechanical stress may contribute to HOA; some studies have reported an association between HOA and intensive manual activities, particularly affecting the dominant hand [14]. Similarly, the roles of smoking and alcohol are unclear, with conflicting results likely influenced by confounding lifestyle and socioeconomic factors [15].

Metabolic comorbidities, particularly cardiovascular diseases and diabetes, have also been associated with HOA. For example, the Framingham Heart Study demonstrated a significant association between symptomatic HOA and incident coronary heart disease (OR 2.26) [16]. Diabetes has also been linked to increased hand pain and functional impairment, especially in erosive forms, although its association with radiographic HOA remains inconsistent [17].

Clinical and paraclinical features

HOA shows marked clinical heterogeneity, ranging from asymptomatic forms to painful and disabling disease. Pain is typically mechanical but may present inflammatory features in erosive forms. Functional impairment is common and may significantly affect daily activities. Validated tools such as the AUSCAN index and the Functional Index for Hand Osteoarthritis (FIHOA) are useful for assessing symptoms and functional impact [18].

Characteristic deformities, including Heberden’s and Bouchard’s nodes and thumb base osteoarthritis (rhizarthrosis), reflect structural progression. Erosive HOA represents a more aggressive phenotype, characterized by inflammatory flares and faster progression.

Radiography remains the reference method for diagnosis and structural assessment using the K&L grading system [19], although it is limited in early disease detection. Ultrasound provides additional value in detecting osteophytes and synovitis [20], while MRI is mainly reserved for research settings due to its ability to assess early structural and inflammatory changes. Laboratory tests are not diagnostic but help exclude other conditions, such as inflammatory or metabolic arthropathies.

Therapeutic management

The management of HOA remains primarily symptomatic and individualized. Non-pharmacological interventions, including hand exercises and orthoses, constitute the cornerstone of treatment. Pharmacological management relies mainly on analgesics and nonsteroidal anti-inflammatory drugs (NSAIDs), particularly topical formulations, which are considered first-line therapy due to their favorable safety profile [21]. Low-dose systemic corticosteroids may be used in inflammatory or erosive forms, while symptomatic slow-acting drugs such as chondroitin sulfate provide modest benefit [22].

To date, no conventional disease-modifying therapies or biologics have demonstrated significant efficacy. Intra-articular injections may provide temporary relief, whereas emerging approaches such as platelet-rich plasma require further validation. Surgical treatment is reserved for severe cases refractory to conservative management [21].

Metabolic syndrome and its association with hand osteoarthritis

MetS is a cluster of metabolic abnormalities, including central obesity, dyslipidemia, hypertension and impaired glucose metabolism, and is widely recognized as a major cardiometabolic risk marker rather than a distinct disease. Its prevalence varies considerably across populations, ranging from less than 10% in some Asian countries [23] to over 40% in Middle Eastern cohorts [24], with intermediate rates reported in North Africa, particularly in Tunisia (31.2%) [25] and Morocco (22-35%) [26]. In our study, the prevalence of MetS was 30.3%, consistent with regional data.

MetS is frequently associated with OA, with prevalence reaching up to 60% in affected patients. Conversely, individuals with OA have an increased risk of developing MetS, suggesting a bidirectional relationship [4]. Several mechanisms have been proposed to explain this association. Hypertension may impair subchondral bone perfusion, leading to cartilage degeneration. Obesity and dyslipidemia contribute through chronic low-grade inflammation mediated by adipokines such as leptin, adiponectin, and resistin. In addition, hyperglycemia and insulin resistance may promote oxidative stress and accumulation of advanced glycation end-products, leading to cartilage damage and chondrocyte dysfunction [3]. These findings support the concept of “metabolic osteoarthritis,” particularly relevant in non-weight-bearing joints such as the hand.

Epidemiological data on the association between MetS and HOA remain heterogeneous. Cross-sectional studies, such as the NEO [27] and METAFIB-OA studies [28], have generally reported a significant association, even after adjustment for BMI. In contrast, longitudinal studies such as Framingham [29] and CASHA [30] have yielded inconsistent results, possibly due to differences in study design, follow-up duration, and adjustment for confounding factors.

In our study, MetS was significantly associated with symptomatic HOA, as well as with several of its individual components, particularly hypertension, diabetes, and abdominal obesity. After multivariate adjustment, only MetS and hypertension remained independently associated with symptomatic HOA.

Overall, while current evidence supports a relationship between MetS and HOA, inconsistencies in longitudinal data highlight the need for large prospective studies to better clarify its role in disease onset and progression.

Limitations of the study

This study has several limitations that should be acknowledged. Being a single-center study may limit the generalizability of the findings to broader populations. The sample size, although sufficient to detect significant associations, remains relatively modest, particularly for subgroup analyses. The lack of assessment of inflammatory biomarkers or adipokines limits deeper exploration of the underlying pathophysiological mechanisms. The absence of longitudinal follow-up also prevents evaluation of disease progression over time. Despite standardized radiographic assessment, potential inter-observer variability may have introduced a minor degree of bias. The 12-year recruitment period (January 2013 to December 2024) represents an additional limitation, as changes over time in clinical practice, diagnostic approaches, imaging techniques, and patient characteristics may have influenced the observed findings.

Finally, potential collinearity between MetS and its individual components should be considered when interpreting the multivariable regression results. Since MetS is defined by the presence of at least three of five metabolic criteria, some of which overlap with the individual variables included in the analysis, such as hypertension and hyperglycemia/diabetes, simultaneous inclusion of MetS and its components may introduce multicollinearity and affect the precision and interpretation of the estimated associations. Therefore, the reported ORs should be interpreted within the context of the specified regression model and should not necessarily be considered evidence of completely independent effects of each metabolic variable.

## Conclusions

Our findings support a significant association between MetS and symptomatic HOA in postmenopausal women, supporting the concept of a metabolic phenotype of the disease. MetS and hypertension were independently associated with symptomatic HOA, while metabolic abnormalities were also associated with greater radiographic severity.

Although no overall relationship was observed between HOA and BMD, lower bone density and vertebral fractures were associated with more severe disease. These results highlight the importance of comprehensive cardiometabolic and bone health assessment, as well as the need for multidisciplinary management and further large-scale prospective studies to clarify underlying mechanisms. 
